# Unlocking therapeutic potential: exploring cross-talk among emerging nuclear receptors to combat metabolic dysfunction in steatotic liver disease

**DOI:** 10.1038/s44324-024-00013-6

**Published:** 2024-07-03

**Authors:** Milton Boaheng Antwi, Ariann Jennings, Sander Lefere, Dorien Clarisse, Anja Geerts, Lindsey Devisscher, Karolien De Bosscher

**Affiliations:** 1 Translational Nuclear Receptor Research, UGent Department of Biomolecular Medicine, VIB Center for Medical Biotechnology, Ghent, Belgium; 2 Liver Research Center Ghent, Ghent University, Ghent University Hospital, Ghent, Belgium; 3 Hepatology Research Unit, Department Internal Medicine and Pediatrics, Ghent University, Ghent, Belgium; 4 Gut-Liver Immunopharmacology unit, Department for Basic and Applied Medical Sciences, Ghent University, Ghent, Belgium; 5 Department of Medical Sciences, The University of the West Indies, St. Augustine, Trinidad and Tobago; 6 Cancer Research Institute Ghent (CRIG), Ghent, Belgium

**Keywords:** Biochemistry, Endocrinology, Gastroenterology

## Abstract

Nuclear receptors (NRs) regulate cellular processes and serve as key targets in treating metabolic dysfunction-associated steatotic liver disease (MASLD) and steatohepatitis (MASH). Their ability to interact and influence each other’s signaling pathways introduces a complex yet underexplored dimension in the pharmacotherapy of MASLD and MASH. This review delineates the emerging NRs in this field—estrogen-related receptor alpha (ERRα), glucocorticoid receptor (GR), estrogen receptor alpha (ERα), liver receptor homolog-1 (LRH-1), and vitamin D receptor (VDR)—and their interplay with established NRs, including peroxisome proliferator-activated receptors (PPARα, PPARβ/δ, PPARγ), farnesoid X receptor (FXR), liver X receptors (LXR), hepatocyte nuclear factor 4α (HNF4α), and thyroid hormone receptor beta (THRβ). We discuss their collective impact on hepatic lipid metabolism, inflammation, fibrosis, and glucose homeostasis. We explore recent findings on dual NR crosstalk, via direct and indirect mechanisms, and discuss the potential of targeting receptor pathways using selective agonists, inverse agonists, antagonists, or specific modulators to combat MASLD and MASH. Elucidating NR interactions opens up new avenues for targeted therapies, emphasizing the critical need for further research in the evolving field of hepatology.

## Introduction

Nuclear receptors (NRs) are transcription factors that play a crucial role in various cellular processes, physiology, inflammation, development, homeostasis, and pathophysiology^1^. Almost 40 years ago, estrogen receptor (ER) was the first NR biochemically identified, and glucocorticoid receptor (GR) was the first NR cloned^2^. Since then, researchers have identified 48 nuclear receptors in humans and 49 in mice that can be activated or inactivated by small molecules, making them a major drug target^1^. Within the superfamily, one can distinguish orphan NRs with no identified natural ligand yet, endocrine NRs that have hormones as endogenous ligands, and NRs that are former orphans^3^. NR ligands include nutrients, vitamins, hormones, and synthetic molecules like exogenous fatty acids, vitamin D3, dexamethasone, and enzalutamide, respectively^4^.

**Fig. 1:**
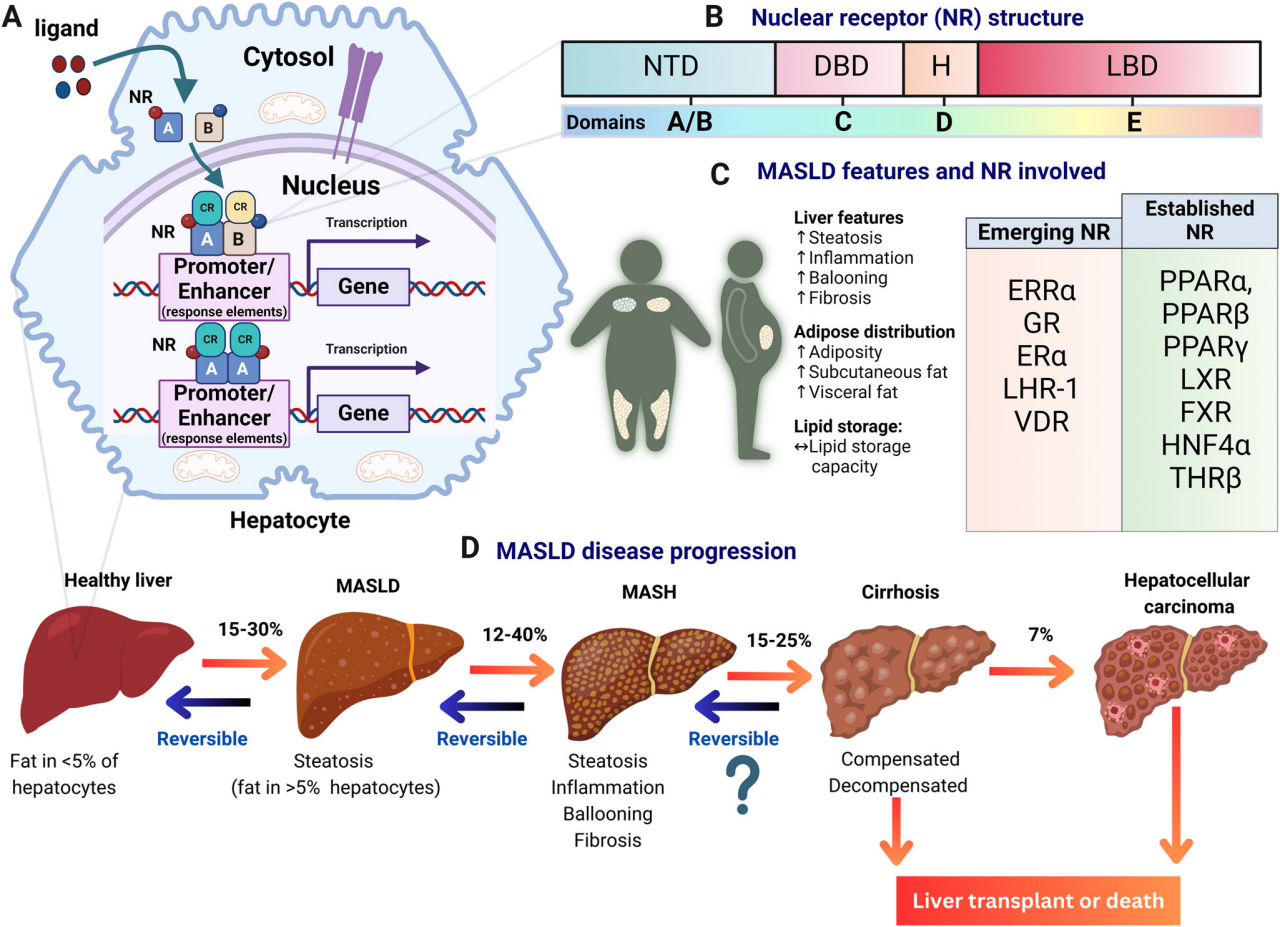
Overview of known and emerging nuclear receptors as targets in metabolic dysfunction-associated liver diseases (MASLD). **A** Representation of nuclear receptors (NRs) bound to DNA response elements in a liver cell. NRs, such as, e.g., peroxisome proliferator-activated receptors (PPARs), liver X receptors (LXRs), and others, can form dimers (homo- or heterodimers with retinoid X receptor [RXR]) to regulate gene expression. NRs are depicted as squares A and B, red and blue circles represent respective ligands, cyan and yellow spheres represent recruited coregulators (CR). **B** General domain structures of nuclear receptors, including domains A/B (containing the transactivation domain), C (DNA-binding domain), D (hinge region), E (ligand-binding domain), and sometimes F (variable C-terminal domain, not depicted here). **C** Hallmarks of MASLD, formerly known as non-alcoholic fatty liver disease (NAFLD), along with a list of nuclear receptors involved in fatty liver disease and potential targets for therapeutic intervention. Key nuclear receptors discussed in this review include estrogen-related receptor alpha (ERRα), glucocorticoid receptor (GR), estrogen receptor (ER), liver receptor homolog-1 (LRH-1), and vitamin D receptor (VDR), alongside established nuclear receptors such as peroxisome proliferator-activated receptors (PPARs), farnesoid X receptor (FXR), liver X receptors (LXRs), pregnane X receptor (PXR), hepatocyte nuclear factor 4α (HNF4α), and thyroid hormone receptor beta (THRβ). **D** Illustration depicting the stages of liver disease progression, starting from fatty liver (steatosis), progressing to steatohepatitis, liver fibrosis, cirrhosis, and potentially hepatocellular carcinoma (HCC).

When inactive, NRs can be located in the cytoplasm or in the nucleus^1^. In the latter case, they may be bound to DNA response elements but are kept inactive due to complex formation with a corepressor. When NRs bind as dimers (either homo- or typical heterodimers with retinoid X receptor [RXR]) (Fig. 1A) to their DNA response element, they can adopt different conformations^5^. Ligand binding via the ligand-binding pocket allows for the release of corepressors and the recruitment of coactivators, which can activate transcription and steer various biological processes^5^.

NRs are classified into subfamilies (NR0-NR6) based on their DNA-binding properties^4,5^. Their structure comprises five domains—A/B, C, D, E, and F (Fig. 1B). The N-terminal A/B domains include the first transactivation domain (AF-1) enabling ligand-independent coregulator interactions^4,5^. The C domain, positioned after the A/B domains, consists of a conserved DNA-binding domain (DBD) with two zinc finger binding motifs and harbors a dimerization function^4,5^. The D domain, or hinge region, is a short, flexible linker that can contain a nuclear localization signal. The E domain, or ligand-binding domain (LBD) includes the ligand-binding pocket, the second transactivation domain (AF-2) allowing for ligand-dependent coregulator interactions and residues crucial for the dimerization of some NRs that form heterodimers^4,5^. Lastly, some NRs have a highly variable C-terminal domain (CTD) known as the F domain, of which the function is not yet fully known^4,5^.

Although NRs can regulate gene expression independently, they can interact with other NRs, in a process known as “dual NR crosstalk”^5^. This allows the expansion and additional modulation of each receptor’s transcriptional response repertoire^5^. The crosstalk mechanisms at the DNA level can be subdivided based on whether the nuclear receptors interact physically^5^. This phenomenon can be exploited for therapeutic purposes by direct or indirect targeting via dual ligand-based approaches^5^.

One disease in which NR targeting is a key drug strategy is metabolic dysfunction-associated steatotic liver disease (MASLD) (Fig. 1C), formerly known as non-alcoholic fatty liver disease (NAFLD)^6^. MASLD is characterized by excessive fat accumulation in the liver, which is linked to metabolic conditions such as obesity and type 2 diabetes mellitus^7,8^. If left untreated, it can progress to an inflamed state called metabolic-associated steatohepatitis (MASH) and further to liver fibrosis, cirrhosis, and hepatocellular carcinoma (Fig. 1D)^7,8^. Alarmingly, the prevalence of MASLD in the adult population worldwide is at 30% and is still rapidly increasing^9^. Recently, resmetirom, a drug targeting the thyroid hormone receptor beta (THRβ) NR, has become the first FDA-approved drug for MASLD, following positive results from the phase III MAESTRO-NASH trial^10^.

Patients suffering from MASLD represent a heterogeneous group, and the development of complementary therapeutic approaches, allowing therapy tailoring based on the disease profile, remains an unmet need^11^. Furthermore, due to the complex multifactorial nature of MASH, which is frequently associated with metabolic comorbidities, the likelihood increases that a combination of different drugs targeting different pathological pathways may become highly relevant to complement or to replace, e.g., in case resistance would emerge, a monotherapy^11^.

Many nuclear receptors have been identified to play a role in MASLD. This review delineates the roles of emerging NRs in MASLD and includes their potential interplay with established NRs (Fig. 1C). We discuss the latest discoveries on 10 dual NR crosstalking events or interactions, and the potential use of selective agonists, inverse agonists, antagonists, and pathway modulation to combat MASLD and MASH (Table 1).

**Table 1:** Interactions and potential therapeutic implications of nuclear receptors in metabolic dysfunction-associated steatotic liver disease (MASLD)

Number	Receptors involved	Putative Crosstalk interaction mechanism	Single ligand/ modulator targeting both NR	Signaling pathways affected in the liver	MODEL/effect of dual NR targeting in the MASLD and MASH context
1	PPARs (PPARα, PPARβ, PPARγ)	Indirect	Lanifibranor	FA breakdown Downregulation de novo lipogenesis Anti-inflammatory effects Glucose homeostasis	Improved MASLD, MASH and fibrosis in various MASLD mice models. Awaiting results of lanifibranor Phase 3 clinical trials.
2	PPARα and ERRα	Indirect and/or direct	Respective ligands affect reciprocal receptor levels in non-hepatocyte systems	ERRα serves as a rheostat for PPARα activity	Not yet investigated
3	PPARα and GR	Indirect and/or direct	GR agonists can affect PPARα receptor levels	Anti-inflammatory effects FA breakdown Gluconeogenesis	Not yet investigated
4	PPARα and LXR	Indirect	None	Induction of lipogenesis Suppression of SREBP1c Reduction of steatosis	Not yet investigated
5	FXR and LXR	Indirect	Acanthoic acid Sesamol	Anti-inflammatory effect Reduced lipid accumulation	Improved MASLD and fibrosis in mice model
6	PPARα and FXR	Indirect	Diosgenin	Regulation of hepatic lipid metabolism FAO upregulation Regulation of hepatic de novo lipogenesis Regulation of hepatokine FGF21	ApoE-deficient mice Rats fed with HFD underwent RYGB bariatric surgery. Patients who underwent RYGB bariatric surgery.
7	LHR1 and GR	Indirect	None	Largely unexplored (LRH-1 regulates glucose and lipid metabolism and intestinal inflammation by controlling GC synthesis)	Not yet investigated
8	HNF4α and GR	Indirect	Nonsteroidal GR antagonist FX5	FX5 suppressed gluconeogenic genes FX5 treatment improved glucose homeostasis and ameliorated hyperglycemia in T2DM mice	Liver-specific GR-KO mice on a high-fat diet Diabetic mouse models such as db/db mice and HFD/STZ-induced
9	VDR and HNF4α	Indirect and/or direct	Oleanolic acid	Normalized fasting serum glucose, insulin levels, insulin resistance, and decreased intrahepatic TG content Oleanolic acid upregulates both NR	HFD-fed mice with a VDR hepatic-KO
10	THRβ and ERRα	Indirect	None	Upregulates FAO Enhances OXPHOS	Not yet investigated

This table provides an overview of the involvement of nuclear receptors (NRs) in MASLD, along with their mode of crosstalk interaction (if known or studied so far), if a single targeted ligand or modulator has been described, affected signaling pathways in the liver, and the effects of dual NR targeting, if known, in the context of MASLD. It serves as a comprehensive reference for understanding the complex interplay between NRs and their therapeutic implications in metabolic liver diseases.

## Emerging nuclear receptors in MASLD

### Estrogen-related receptor alpha (ERRα)

ERRα (*NR3B1*), sometimes referred to as “energy-regulated receptor”, is a constitutively active orphan nuclear receptor that is primarily regulated by physiological cues like cold and starvation and through coregulators such as PPARγ coactivator (PGC)-1α^12^. It controls mitochondrial energy pathways, including fatty acid β-oxidation (FAO) and oxidative phosphorylation. Developing ERRα agonists has been challenging due to ERRα’s small LBD, making inverse agonists with smaller structures more feasible^13^. Inhibition of ERRα showed promise to impede MASLD features and progression in preclinical models, though not yet in clinical trials^14–17^.

A study conducted in three MASLD models using polyamide ERR-PA, which inhibits ERRα by binding to its response elements, was effective in curtailing the development of MASLD and MASH^14^. ERR-PA reduced de novo lipogenesis (DNL) gene expression (e.g., *Fasn, Acc*) and lowered triglyceride (TG) and plasma glucose levels. In another study, ERRα KO mice exhibited resistance to weight gain, reduced white adipose tissue, and hepatic fat accumulation, and improved insulin sensitivity and glucose uptake when subjected to HFD^15^. The knockout (KO) phenotype coincided with repression of lipogenic genes such as *Fasn, Acc1*, and *Scd1*, although surprisingly, these mice also exhibited reduced FGF21, a protein protecting against hepatic lipotoxicity^18^, and an enhancer of mitochondrial respiration rates^15^.

ERRα acting downstream of estrogen/ERα signaling formed the basis of an observed sex-difference in hepatic very low-density lipoprotein (VLDL) secretion. Higher hepatic ERRα expression in female mice contributed to the formation and release of hepatic VLDL^19^. Reduced hepatic VLDL-TG secretion was linked to hepatosteatosis, contributing to MASH development^19^. Deleting ERRα in the liver also exacerbated MASLD and insulin resistance in mice^19^. In agreement, a novel ERRα agonist JND003 ameliorated insulin sensitivity and MASLD by activating ERRα downstream target genes, an effect not observed in ERRα-deficient mice^20^.

In terms of drug development, a potent pan-ERR agonist, SLU-PP-915, was synthesized to target all subtypes of ERRs and awaits evaluation in MASLD models^21^. These findings underscore the need for more ERRα research, particularly regarding its anti-fibrotic and anti-inflammatory roles, and the development of clinical-use compounds.

### Glucocorticoid receptor (GR)

Glucocorticoids (GCs) can target GR (*NR3C1*) and are efficacious drugs in treating various inflammatory disorders^22^. In a fed state, besides supporting genes involved in hepatic glucose uptake^23^, GR also drives genes connected to DNL, VLDL production, hepatic fatty acid uptake, and FAO^24^.

Chronic stress or prolonged exposure to high levels of GCs is implicated in MASLD development in man^25^. Dexamethasone, a widely used GR-targeting drug for over 200 health conditions, induces hepatic lipid accumulation in mice, primarily via CD36 upregulation^26^. GR inhibition using mifepristone (RU486) or knockdown mitigated the effect by specifically targeting CD36 upregulation^26^. Thus, strategies that inhibit the GR/CD36 axis can potentially prevent or alleviate GC-induced hepatic lipid metabolism disorders^26^. Alternatively, a dexamethasone-induced fatty liver in mice was reduced by inhibiting fat mass and obesity-associated (FTO) protein, involved in adipogenesis and tumorigenesis^27^.

The GR ligand CORT118335 prevented and reversed hepatic lipid accumulation in mice on HFD^28^. Its unique blend of partial GR agonism and antagonism benefits the liver by upregulating GR target genes involved in VLDL production and secretion, alongside enhancing whole-body FAO in extrahepatic tissues^28^. CORT125385 similarly reduced liver steatosis in mice by modulating GR^29^.

Besides GR ligands, overexpression of the microRNA miR-192-3p ameliorated microvesicular steatosis and insulin resistance, but this effect was ablated by GR reactivation^30^. Other evidence linking GR activity to MASLD was derived from a MASLD-prone mouse model caused by hepatocyte-specific overexpression of the enzyme converting inactive to active GCs, 11β-Hydroxysteroid dehydrogenase 1 (11β-HSD1)^31^. Subsequent GR nuclear translocation and GR phosphorylation resulted in the upregulation of key lipogenic regulatory proteins FAS, SCD1, SREBP1, and ACC1^31^.

These studies suggest that controlled targeting of GR signaling in the liver may be a promising therapeutic strategy for treating MASLD and MASH. Still, because endogenous glucocorticoids are involved in a well-balanced circadian rhythm^32^, and given a dysregulated circadian rhythm not only disturbs immune functioning but also contributes to MASLD and cardiovascular disorders, GR-targeting agents should be either novel generation molecules with a narrow activity spectrum or exhibit a unique profile following crosstalk mechanisms with other nuclear receptors, to only target those activities that are of benefit within the specific pathology of MASLD^33,34^. An important aspect to potentially reduce undesired disturbances to our internal body clock caused by drugs that target the GR protein is to consider the impact of drug administration time.

### Estrogen receptor alpha (ERα)

Estrogens, which bind to ERα (*NR3A1*), are more prominent in females and found in various tissues, including white adipose tissue, liver, and muscle^35^. Three types of ERs mediate the action of estrogen: ERα, ERβ, and G protein-coupled ER on the plasma membrane^35^. In examining gender disparities, ERα is crucial in MASLD progression, lipid metabolism, and inflammation^35,36^.

Premenopausal women exhibit greater insulin sensitivity than males, a difference attributed to lower ER protein expression^37^. Despite the relatively low ERα protein expression in males, ERα ablation in male HFD-fed mice increased adiposity, glucose intolerance, hepatic steatosis, and inflammatory gene expression in epididymal WAT and liver compared to wild-type controls^38^. Therefore, ERα is vital for optimal immunometabolic function, sex-specific reprogramming of liver metabolism, and metabolic flexibility, which is crucial for counteracting the adverse effects of HFD^38–40^.

A study focusing on ERα’s mechanistic role revealed that regulating Adropin, dependent on ERα, could be a factor in the observed sex differences^39^. In female ovariectomized mice fed with HFD, estrogen deficiency-induced macrophage recruitment, fibrogenesis, accelerated hepatic steatosis, and MASH development^41^. ERα knockdown exacerbated monocyte infiltration and increased proinflammatory macrophages by upregulating CCR2 gene expression^41^. Antagonizing CCR2 effectively improved MASH, endoplasmic reticulum stress, and insulin resistance in obese female mice with ERα knockdown^41^.

Recently, marked ERα-binding was identified within the patatin-like phospholipase domain-containing 3-(PNPLA3) p.I148M variant enhancer^42^. Hepatic PNPLA3, a key factor in inherited MASLD variability, exhibited higher expression in obese women than men and correlated with estrogen levels in mice. Furthermore, in human hepatocytes and liver organoids, PNPLA3 can be induced by ERα agonists^42^.

Taken together, the above results highlight that the complex interplay between estrogen, ERα, and liver health is differently regulated in males and females, an important point to consider for MASLD therapeutic interventions and clinical trials.

### Liver receptor homolog-1 (LRH-1)

LRH-1 (*NR5A2*) regulates diverse biological functions, such as the cell cycle and steroid homeostasis^43^. It is a phospholipid-bound TF that plays a pivotal role in the liver, particularly in glucose, cholesterol, and bile acid metabolism^44^. Similar to other NRs, the transcriptional activity of LRH-1 largely hinges on the recruitment of coregulators that interact with the AF-2 domain of LRH-1^44^.

In the context of MASLD, LRH-1 is downregulated in human patients with simple hepatic steatosis or MASH^45^. Studies using SUMOylation-defective mutants of LRH-1 in mice have shown increased hepatic TG content and DNL, making them more susceptible to steatosis, inflammation, and fibrosis fed with HFD than wild-type mice^46^. The LRH-1 K289R mutant induces oxysterol-binding protein-like 3 (Osbpl3), which further activates sterol regulatory element-binding protein 1 (SREBP-1), a TF-driving DNL and known to be elevated in MASLD^46^. Hepatic LRH-1 KO in mice, independent of HFD, increased TGs, steatosis, fibrosis, liver injury, and glucose intolerance, which improved with the expression of wild-type human LRH-1^47^. Additionally, there was a decrease in FAO and altered gene pathways in lipid storage and phospholipid metabolism^47^. Disrupted phospholipid composition, caused by reduced arachidonoyl (AA) phospholipids, was attributed to the repression of critical AA biosynthesis genes and identified LRH-1 targets Elovl5 and Fads2^47^.

The search for an effective LRH-1 agonist is ongoing^48^. Alternatively, the epigenetic remodeler MORF4-related gene on chromosome 15 (MRG15) could be targeted for the treatment of MASH due to LRH-1’s role in mediating rhythmic MRG15 recruitment to lipid synthesis genes^48,49^.

### Vitamin D receptor (VDR)

VDR (*NR1I1*) binds active vitamin D and regulates over 900 genes, many of which are associated with bone metabolism and immune functions^50,51^. Within the nucleus, the VDR-vitamin D complex heterodimerizes with RXR and binds to VDR response elements (VDRE) to modulate gene expression^52^.

In MASLD, activated VDR has notable anti-inflammatory effects, exhibits anti-fibrotic properties, and improves steatosis and insulin sensitivity in liver cells^53^. Some multivariate analysis in biopsy-proven MASLD patients has linked vitamin D deficiency and gene polymorphisms to advanced fibrosis and its severity^51,54^, although this association was not observed in another study^55^. Studies also highlighted the association of VDR gene polymorphisms, particularly BsmI, FokI, TaqI, and ApaI, with the severity of liver cirrhosis and MASLD in pediatric patients^56,57^. In vitro experiments examining VDR single nucleotide polymorphisms (SNPs), like the TaqI SNP, notably reduced VDR expression, increased fibroblast proliferation, and upregulation of extracellular matrix proteins^58^. Furthermore, the binding of vitamin D to membrane-bound VDR can activate PKC and ERK/MAPK signaling pathways, influencing the production of pro- or anti-inflammatory mediators depending on the stimuli added^51^.

A protective role of vitamin D-VDR signaling in hepatic steatosis development has been substantiated in vivo^59–62^, and includes organ crosstalk mechanisms. Female adipose-specific VDR knockout mice exhibit increased visceral adipose tissue, hepatic lipid content, and upregulation of genes related to fatty acid transport, synthesis, and oxidation^59^. Rats treated with vitamin D on an HFD showed decreased liver enzymes and reversed steatosis, alongside improved gut microbiota^60^. Similarly, MASLD rat models and oleic acid-treated HepG2 cells showed improved hepatic steatosis following vitamin D treatment^60^. Studies on human progenitor hepatic cells showed that vitamin D3 reduces lipotoxicity^63^. Collectively, these findings underscore the important role of VDR and vitamin D in the pathophysiology and potential treatment strategies for MASLD.

## Crosstalk between emerging and established NR to ameliorate MASLD and MASH

### Crosstalk between PPARα, PPARβ, and PPARγ

Peroxisome proliferator-activated receptors (PPARs), a family of NRs that can heterodimerize with RXR, include three isoforms with distinct ligand specificity and features: PPARα (*NR1C1*), PPARβ/δ (*NR1C2*), and PPARγ (*NR1C3*)^64,65^. These isoforms are co-expressed across various organs and tissues^64^. PPARs are receptors for various endogenous lipids, including unsaturated, monounsaturated, polyunsaturated fatty acids, and exogenous synthetic compounds^64^.

The role of PPARs in liver injury has been extensively documented in preclinical and clinical studies^64^. They are recognized as effective targets for treating various metabolic syndromes, including dyslipidemia, diabetes mellitus, and MASLD^64^. PPARα is highly expressed and active in the liver, facilitating TG turnover, fatty acid uptake, β-oxidation, and modulating inflammation. PPARβ contributes to lipid metabolism, glucose homeostasis, and inflammation, while PPARγ, predominantly expressed in adipose tissue, is crucial for adipocyte differentiation, adipogenesis, lipid metabolism, and anti-inflammatory properties^66–68^. Notably, PPARγ also plays a role in the sexual dimorphism observed in MASLD in mice^67,69^.

Some PPAR drugs were developed targeting more than one isoform. Elafibranor, a dual PPARα/δ agonist, initially showed signs of benefiting MASLD^70^. However, its Phase 3 interim analysis clinical trials (RESOLVE-IT) for MASH failed to demonstrate a statistically significant effect on the primary endpoint of MASH resolution without worsening of fibrosis. Currently, Elafibranor is being investigated for primary biliary cholangitis, and its Phase 3 clinical trials (ELATIVE) show promising results^71^. Saroglitazar, a PPARα/γ ligand, is used for treating type II diabetes and has been shown to improve dyslipidemia, liver enzymes, and fibrosis in patients with MASLD and MASH^72^.

A notable development is Lanifibranor, a pan-PPAR agonist currently in phase III trials (NATIVE-3) for MASH with fibrosis stages F2 and F3^73–75^. Lanifibranor exhibits marked macrophage modulatory effects and has a more potent impact on inflammation and disease progression as compared to selective PPAR agonists^74,75^. It activates all PPAR subtypes with varying potencies and efficacies^76^. The plethora of data highlights the relevance of ligand diversity and the potential of PPARs as therapeutic targets in MASLD and MASH management.

### Crosstalk between PPARα and ERRα

Literature findings support a close functional interaction between PPARα and ERRα^77–80^. Both NRs are part of an intricate network regulating metabolism^80^. For example, in the mouse liver, deficiency of hepatocyte E3 ligase Fbxw7 was found to relieve ERRα degradation, which in turn reduced PPARα activity and suppressed hepatic catabolism^77^. A recently identified NR crosstalk mechanism indicates a role for ERRα as a rheostat for PPARα activity, involving a PGC1α-promoted interaction between both receptors in the hepatocyte nucleus. In line, PPARα agonists supported the recruitment of ERRα at promoter and enhancer regions of PPARα-controlled target genes in non-fasted mouse livers^81^. Further research is needed to explore the potential benefits of using a combination of PPARα and ERRα ligands in vivo.

### Crosstalk between PPARα and GR

PPARα and GR exhibit similar and complementary functions in liver metabolism, particularly concerning carbohydrate and fat metabolism^82^. They exquisitely orchestrate catabolic and anabolic pathways by collaboratively regulating key genes that are essential for maintaining healthy blood glucose levels. Molecular mechanisms of how PPARα and GRα coordinately support the breakdown of fatty acids and drive gluconeogenesis during physiological fasting while oppositely organizing glucose and fat energy depots in fed states are currently being unraveled^82–84^. The interplay between PPARα and GR was demonstrated to inhibit NF-κB-driven expression, effectively downregulating inflammatory responses^83^. Despite the individual role these NRs play in MASLD, the simultaneous targeting of PPARs and selective GR modulation in MASLD may hold potential but have not yet been explored in mouse models or clinical settings^64,82,84^.

### Crosstalk between PPARα and LXR

LXRs play a crucial role as regulators of sterol regulatory element-binding proteins (SREBPs), which are central to DNL (FASN, SCD1, and SREBP1c), cholesterol synthesis, anti-inflammatory activities, and immunity^85^. The LXRα (*NR1H3*) isoform is predominantly expressed in the liver and, to a lesser extent, in the kidney, intestines, and adipose tissue, while the LXRβ (*NR1H2*) isoform is ubiquitously expressed^85^. Because LXRs were linked to the exacerbation of MASH^86^, synthetic agonists are of little use due to their propensity to stimulate DNL, shifting the focus instead to inverse agonists^87–90^.

The interconnection between PPARs and LXRs is well-established, given their recognition of similar response elements and shared target genes^91^. Recent studies have shown potential in targeting steatosis by disrupting the phosphorylation at Ser196 in LXRα^92^. Tandem research in murine primary hepatocytes and mouse livers has indicated that PPARα can suppress the SREBP1c promoter by inhibiting LXR signaling, and vice versa, that LXRs suppress lipid degradation-supporting gene promoters through inhibition of PPAR signaling^93^. The mechanism was reported to involve ligand-induced shifts in PPARα/RXR versus LXRα/RXR heterodimer formation^93^. Furthermore, when combined with insulin, LXR and PPARα agonists collaboratively induce the expression of lipogenic genes such as FAS and acetyl-CoA carboxylase 1 (ACC1)^93,94^. Taken together, the data highlight the intricate relationship and potential joint therapeutic entry points involving these NRs.

### Crosstalk between FXR and LXR

FXR (*NR1H4*), with four isoforms and high expression in the liver, primarily regulates bile acid homeostasis and biosynthesis from cholesterol^95^. Beyond this, FXR influences lipid metabolism, glucose regulation, oxidative stress, inflammation, and microbiome dynamics^95^. FXR agonists protect against MASLD and reduce lipid metabolism, fibrosis, and MASH through bile acid-dependent mechanisms^96–98^. Despite positive outcome data in the REGENERATE trial^98^, the FXR agonist obeticholic acid was not granted FDA approval for MASLD.

Recent studies have shed light on the interplay between FXR and LXR^99^. Acanthoic acid, exhibiting anti-inflammatory and hepato-protective effects, reduced lipid accumulation in mice on an HFD, lowered TG and SREBP-1 levels, and improved hepatic fibrosis markers through the activation of LXR and FXR signaling^99^. This activation escalated the AMPK-SIRT1 signaling within the FXR-LXR axis, a mechanism proving effective in mitigating MASLD^99^.

The lignan Sesamol emerged as a potential regulator of hepatic fibrosis, inhibiting fibrogenesis, autophagy, and inflammation^97^. This regulation is achieved via the FXR/LXR axis-mediated inhibition of autophagy, which is crucial in the crosstalk between hepatic stellate cells (HSCs) and macrophages, highlighting the complex interdependencies and therapeutic potential of the jointly triggered FXR and LXR pathways in liver disease management^97^. Concerning FXR/LXR crosstalk, an important consideration is the inherent metabolic difference between mice and humans in terms of cholesterol transport and handling^100^. Cholesteryl ester transfer protein (CETP) facilitates the transfer of cholesteryl esters and triglycerides across lipoproteins in humans, playing an important role in lipid metabolism and the reverse cholesterol transport route. Its absence in mice and rats implies that murine high-density lipoprotein (HDL) cholesterol is not easily transferred to other lipoproteins, resulting in higher HDL levels and a different lipoprotein profile as compared to humans.

### Crosstalk between PPARα and FXR

The interplay between PPARα and FXR plays an essential role in regulating hepatic lipid metabolism, FAO, hepatic lipogenesis, and the regulation of hepatokine FGF21^101,102^. Additionally, FXR can enhance the expression of hepatic carboxylesterase 1, aiding in the release of fatty acids and thus upregulating PPARα expression^103^.

In vivo, the loss of FXR was exacerbated while a PPARα agonist reversed atherosclerosis and hepatic steatosis in ApoE-deficient mice^104^. In male Wistar rats on an HFD, liver fat loss following Roux-en-Y gastric bypass (RYGB) surgery was attributed to increased fasting bile acid levels and the modulation of liver fat oxidation by FXR and PPARα^105^. Notably, intact PPARα signaling was essential for resolving MASLD post-RYGB^105^.

The natural compound Diosgenin has shown efficacy in alleviating MASH by modulation of the FXR-SHP-SREBP1C/PPARα/CD36 pathway in rats fed with HFD and FFA-treated HEPG2 cells^106^. Mechanistically, Diosgenin upregulated FXR and SHP, while downregulating the expression of genes involved in DNL, such as SREBP1C, ACC1, and FASN^106^. Diosgenin also upregulated PPARα gene expression, likely via an FXR-dependent induction of its promoter activity. Lower expression of the fatty acid translocase CD36 by diosgenin involves a role for hepatic FXR, albeit the exact mechanism remains to be resolved^106^. Collectively, while both PPARα and FXR collaboratively influence hepatic functions consistent with reduced lipid accumulation, the exact mechanisms of their interaction warrant further investigation.

### Crosstalk between LHR1 and GR

The crosstalk between LRH-1 and GR in the liver remains largely unexplored. However, LHR-1 in the instestine is known to regulate glucose and lipid metabolism and intestinal inflammation by controlling GC synthesis^107^. LRH-1 plays a critical role in synthesizing GC in the intestinal epithelium^108^. Its absence in the intestinal epithelium causes intestinal inflammation, due to the lack of local GC synthesis^108^. In line, LRH-1 was suggested as a potential marker for GC-impaired responses in ulcerative colitis^109^. Another study highlighted the role of local GC synthesis via LRH-1 in immune evasion within colorectal tumors, suggesting LRH-1’s potential as a therapeutic target^110^. Data from other systems reveal a knowledge gap, particularly regarding the interaction between LRH-1 and GR in the liver. Understanding this crosstalk could be pivotal in developing new therapeutic strategies for MASLD and MASH.

### Crosstalk between HNF4α and GR

Hepatocyte nuclear factor 4α HNF4α (*NR2A1*) is a liver-enriched nuclear receptor whose expression decreases markedly in conditions such as diabetes and MASLD, which are associated with hyperlipidemia^111^. Intriguingly, mice genetically engineered to lack hepatic HNF4α develop MASLD, yet exhibit low blood lipid levels and are atherosclerosis-resistant^112,113^. Similarly, in humans, HNF4α expression diminishes from healthy to MASLD to MASH^114^.

A study of the interaction between HNF4α and GR reveals that at those sites where GR binds within open chromatin, the HNF4α motif is adjacent to glucocorticoid response elements (GRE)^115^. Hence, HNF4α absence remodels the liver GR cistrome and alters GR recruitment^115^. Vice versa, liver-specific GR-KO mice on a high-fat, high-sugar diet showed increased hepatic lipid accumulation concomitant with an induction of SREBP1C and PPARγ and downregulated HNF4α protein levels in line with decreased lipid catabolic gene expression^116^.

Other disease models may show different outcomes of crosstalk mechanisms. Exploring the connection between HNF4α and GR yielded a small, nonsteroidal GR antagonist molecule, 5-chloro-N-[4-chloro-3(trifluoromethyl)phenyl]thiophene-2-sulfonamide (FX5). In diabetic mouse models such as db/db mice and HFD/STZ-induced mice, FX5 repressed the Hnf4a gene, while improving glucose impairment by mitigating gluconeogenesis^117^. This effect was demonstrated in primary murine hepatocytes by a direct mechanism antagonizing GR and an indirect mechanism, partially, through modulation of the GR/HNF4α and GR/HNF4α/miR122-5p signaling pathways^117^. Further investigations need to be conducted to better understand GR and HNF4α crosstalk to aid in drug development targeting both NR to ameliorate MASLD.

### Crosstalk between VDR and HNF4α

VDR and HNF4α engage in notable interaction and crosstalk^118^. Overexpression of HNF4α in HFD-fed mice with a VDR hepatic-KO normalized fasting serum glucose, insulin levels, insulin resistance (measured by HOMA-IR), and decreased intrahepatic TG content^118^. The underlying mechanism for vitamin D-mediated amelioration of MASLD in wild-type mice involves an interaction between activated VDR and HNF4α^118^.

While direct drug targeting of both receptors has not been extensively explored, there is evidence that oleanolic acid can upregulate the expression of these NR, particularly VDR and HNF4α^119^. This upregulation alleviated inflammation and cholestasis in bile duct-ligated rats, indicating potential therapeutic benefits^119^. However, the broader implications of enhancing the expression of both receptors warrant further investigation.

### Crosstalk between THRβ and ERRα

THRβ and ERRα are key regulators of gene expression for numerous mitochondrial pathways in the liver^120^. Research has shown that the thyroid hormone T3 (TH), a ligand for THRβ, increased ERRα expression and activity in a THRβ-dependent fashion^120^. Vice versa, ERRα can also regulate the expression of the THRB1 gene^120^. ERRα was shown to be crucial in enhancing oxidative phosphorylation, triggering the production of tricarboxylic acid cycle metabolites and activation of FAO through the action of TH^120^. The data imply that either hormonal or pharmacologic induction of ERRα expression or activity could improve mitochondrial quality in metabolic disorders. The interplay between THRβ and ERRα in mitochondrial function and lipid metabolism, specifically in the MASLD context, awaits further exploration.

## Concluding remarks and future perspective

The individual activity and interplay among NR in the liver presents a compelling narrative of metabolic regulation and disease modulation of MASLD and MASH. The insights from studying these interactions may offer a deeper understanding of liver physiology and pathology, and may provide a solid foundation for therapeutic interventions. Because many studies rely on rodent models, interspecies variations in nuclear receptor-mediated mechanisms should not be overlooked. Species-specific nuclear receptor-mediated control of CYP450-dependent pathways is one example hereof^121,122^. Species-dependent variations can change the expression of these enzymes, resulting in species-specific drug metabolism. This might lead to variations in drug effectiveness to even inaccurate safety assumptions in certain pathways. To overcome these issues, the use of humanized mouse models, in vitro cell-based systems such as human hepatocytes/organoids, and advanced computational models to predict metabolic processes based on molecular and genetic information may help bridge the gap between animal models and human conditions^123,124^. Acknowledging these differences helps in designing better preclinical studies and potentially in developing more accurate biomarkers for assessing drug efficacy and safety in human populations. While challenging, the complexity of crosstalking NR-dependent networks also represents a treasure trove of opportunities for innovative treatment strategies that could create a momentous impact and ameliorate MASLD. Future research should unravel the mechanisms underpinning NR interactions through cutting-edge technologies. Such endeavors could pave the way for precision medicine strategies that target specific NR pathways.
